# New roles in pharmacy – learning from the All Wales Common Ailments Scheme

**DOI:** 10.1111/ijpp.12231

**Published:** 2015-12-16

**Authors:** Efi Mantzourani, Thomas Gwyn Richards, Mary Louise Hughes

**Affiliations:** ^1^ Cardiff School of Pharmacy and Pharmaceutical Sciences Cardiff University Cardiff Wales

**Keywords:** common ailments service, community pharmacy, expanding role, new services, service evaluation

## Abstract

**Objectives:**

The objective of this study was to explore the perceptions of stakeholders on a national pilot of a new service, the ‘Choose Pharmacy’ Common Ailments Service (CAS) in Wales.

**Methods:**

Methods used were semi‐structured interviews with stakeholders involved in development and delivery of the CAS. Snowball sampling was employed and invites were extended to eight of 13 pharmacies offering CAS in Cwm Taf LHB, the practice managers at two associated general practitioner surgeries and two local and national level commissioners.

**Key findings:**

The benefits of encouraging self‐care by patients were widely recognised in terms of their impact on patients, health professionals and wider society. Although some challenges of introducing a new service were identified, these did not appear to be insurmountable.

**Conclusions:**

CAS was welcomed by stakeholders in terms of its potential benefits. Results are therefore encouraging for policy makers involved in the implementation of other new roles within community pharmacy in the UK and beyond.

## Introduction

Minor or common ailments are defined as acute health complaints, which are self‐limiting or can be treated straightforwardly by patients themselves with minimal medical intervention.[1] In October 2013, the Welsh Government announced the national ‘Choose Pharmacy’ Common Ailment Scheme (CAS), with the aim of making ‘*community pharmacy the first port of call for the provision of advice and where necessary treatment of common illnesses*’.[2] While the benefits of CASs have previously been identified in quantitative terms, few studies have investigated the perspective of those involved in their development and delivery.[3, 4] The aim of this study was to explore the perceptions of stakeholders involved in all stages of the pilot of the All Wales CAS.

## Methods

This study was designed through an interpretivist paradigm.[5] It was based in the Cwm Taf University Health Board area. Eight out of 13 pharmacies offering CAS were invited to participate; this represents a cohort of 25% of the pharmacies piloting the service in Wales. Other stakeholders identified were general practitioner (GP) practice managers working at a local surgery, involved in patient referral, and two key informants i.e. one individual from the local Health Board and one from the Welsh Government involved in developing and commissioning the service. Staff with limited involvement in the scheme (locums and healthcare assistants) were not included in the sampling frame. Purposive and snowball sampling methods were employed.

Approval was received from Cardiff School of Pharmacy and Pharmaceutical Sciences Research Ethics Committee. Signed informed consent was obtained prior to data collection.

Semi‐structured interviews were conducted by an independent researcher, based on a core interview schedule (Appendix 1), with specific prompts for each stakeholder group. The interviews were audio‐recorded and transcribed *ad verbatim*. The anonymised transcriptions were thematically analysed inductively, using constant comparison to reveal overarching themes.

## Results

Eleven stakeholders: seven pharmacists, two GP practice managers and two key informants agreed to participate and were interviewed between February and March 2014. Three themes emerged related to perceptions and attitudes towards the scheme (Table 1).

**Table 1:** Themes related to perceptions and attitudes towards the CAS scheme, and illustrative quotes

Theme	Example quote	Participant
Pharmacist professional image	As pharmacists we get to increase the profile and awareness of what pharmacists can offer. We can offer a whole range of different services and have the clinical skills in place and the facilities in place to offer these services. However, patients often don't come to us because they're not really aware of the underpinning knowledge. Unfortunately a lot of people still see us as tablet dispensers.	Pharmacist 1
	I think the advice that they get with the consultation, because they're speaking one‐to‐one with the pharmacist, I think they benefit from advanced advice compared to what they might get from a counter assistant.	Pharmacist 3
	As the service grows and develops and an awareness of it grows, then hopefully people will be using pharmacies as the first port of call.	Pharmacist 1
Promotion of self‐care	I want it to demonstrate we can successfully move patients from GP appointments into community pharmacy for the pharmacist to treat them and ultimately I want them to be able to move from that into self‐care.	Key Informant 1
	If the only thing you know about treating an illness is to go and see a GP, then you get a prescription and it reinforces your belief that what you should always do is go back and see a GP … [CAS] encourages patients to recognise their symptoms and be able to treat themselves in the future without having to call on the NHS.	Key Informant 2
	The main thing I think is to re‐educate patients that they don't need to use the service; they could just come in and buy something for a minor ailment … self‐care basically.	Pharmacist 4
	We are drowning in primary care with non‐essential things, people who feel that they are unwell rather than are actually unwell.’	Practice Manager 1
	If there were six patients going over there [pharmacy], then that's an hour's worth of consultations from here [surgery] and that would greatly help and reduce our workload.	Practice Manager 2
	[CAS] allows them to see the patients whose blood pressure isn't controlled, allowing them to see that patient earlier than they would have. If you do that enough times you prevent strokes.	Key Informant 2
	The greater benefit for Welsh Government is this one about promoting patients who are able to self‐care … treat themselves earlier, end up not progressing to a point where they are very ill and therefore needing more interventionist or more urgent services that are inevitably more expensive.	Key Informant 2
	What, I don't think we want to do, is push people who will readily buy medication for conjunctivitis and other common ailments into the common ailments system. If patients want to buy them, why should they be treated under the NHS when they don't need to be?	Pharmacist 2
Challenges of introducing a new role	As well as trying to do all the other things they're [pharmacists] trying to do, the dispensing, the DMRs, the MURs and all the other services, they'd have fifty people sitting in the pharmacy wanting minor ailments consultations, so they were really nervous about us doing a big push on launch.	Key Informant 1
	I think, yes it is time consuming, but to actually push pharmacy forward we need to be giving this time to the service … I think it's worthwhile time … in terms of the time you put in for the image of the profession, I think it's important.	Pharmacist 3
	Hopefully it will improve relationships with GPs as well, so we'll improve communication and improve cross‐sector working and again we'll improve on things for the future.	Key Informant 1
	When all the GPs emergency slots were filled, they [receptionists] were then being referred regardless to CAS. Obviously under that system you are going to get an awful lot of inappropriate referrals.	Pharmacist 1
	It's easier for them [receptionists] to make an appointment for somebody, they can do that in seconds, than have to explain the scheme.	Practice Manager 2
	Hopefully it will improve relationships with GPs as well, so we'll improve communication and improve cross‐sector working and again we'll improve on things for the future.	Key Informant 1

CAS, ‘Choose Pharmacy’ Common Ailments Service; DMR, Discharge Medicines Review; GP, general practitioner; MUR, Medicines Use Review; NHS, National Health Service.

### Pharmacist professional image

All stakeholders noted that with the advent of clinical pharmacy services within community pharmacy, patients are accepting of the pharmacy setting for consultations and receiving treatment. However, it was felt there was still a lack of understanding of the role of the modern‐day pharmacist. For the pharmacist stakeholders, additionally CAS was an ideal opportunity to improve the profession's image and to increase public and GP awareness of the services that pharmacy can offer.

### Promotion of self‐care

The majority of the stakeholders' expectations centred on promoting patients' use of pharmacies as the first port of call for minor ailments, ultimately promoting self‐care. Stakeholders from all groups identified CAS as a valuable tool to re‐educate patients and increase their confidence in diagnosing and treating minor ailments themselves, decreasing pressures on GPs. The potential facilitation of patients with more pressing health issues to consult their GP earlier was emphasised. Both key informants noted increased self‐care could reduce health expenditure and more prudent use of NHS resources. However, there were concerns that low [self‐care] rather than high [GP appointments] users of NHS resources would use the scheme, thus counteracting its rationale (Table 1).

### Challenges of introducing a new role

Pharmacists highlighted that even though CAS adds to workload, they recognise it as an important opportunity to promote pharmacy.

Although all pharmacists felt prepared to offer CAS, they perceived that GP receptionists, who make the vast majority of suggestions to patients about entering the scheme, needed more support and training for this role.

Pharmacists and practice managers reported either unchanged or improved interdisciplinary relationships with other stakeholders. Several interviewees recognised that meeting other stakeholders during the development phase increased understanding of each other's roles and this early engagement increased the sense of ownership. It was anticipated that relationships would continue to develop.

## Discussion

Stakeholders viewed CAS as an opportunity to redefine the community pharmacists' image and promote their professional identity. Time and associated workload was considered a barrier; however, this was tempered by a sense that perceived benefits outweighed barriers. Pharmacists in the present study felt they were sufficiently trained, as found elsewhere,[6, 7] but believed that further training may be required for surgery staff.

Almost all interviewees associated the scheme with promoting patients' use of pharmacies as the first port of call for advice and treatment of minor ailments, consistently using the term ‘self‐care’. This contrasts with the finding of an earlier CAS evaluation in England, in which pharmacists did not associate CAS with encouraging self‐care,[6] despite this concept being a high priority item in the UK Government's agenda at that time.[8]

This is an important finding as it perhaps signifies a shift in pharmacists' attitudes towards fostering a culture of self‐care.

### Strengths and limitations

This is the first qualitative review of the All Wales Common Ailments Scheme national pilot. The findings are potentially of interest to many countries, especially those who are looking to enhance self‐care as a means to promote prudent healthcare. While it is acknowledged that the sample size was small, a range of views and perspectives were uncovered, helped by the inclusion of the majority of participating pharmacists in the pilot region as well as the key informants from the Health Board and Welsh Government. Although due to the limited scale of the study, the results cannot be generalised and it cannot be concluded that stakeholders' perceptions regarding CAS reached a point of theoretical saturation, the last interviews unearthed no new concepts. Views of patient and GPs will need to be sought for a more holistic review of the scheme.

## Conclusions

This study explored the pilot All Wales Common Ailments Scheme from the perspectives of those involved in its design and delivery. The service was welcomed by the stakeholders in terms of its potential benefits, namely an improved public image of community pharmacy and a societal shift towards self‐care for minor ailments. Although some challenges of introducing a new service were identified, these did not appear to be insurmountable. It was highlighted that engagement in the early stages of the scheme increased sense of ownership among stakeholders. The service commissioners from the Welsh Government and Local Health Boards must continue to engage with all CAS stakeholders to facilitate the ongoing success of the scheme. Lessons learned from the present study can help policy makers intending to develop and introduce new community pharmacy services, whether in the UK or beyond.

## Declarations

### Conflicts of interest

The Author(s) declare(s) that they have no conflicts of interest to disclose.

### Funding

This research received no specific grant from any funding agency in the public, commercial, or not‐for‐profit sectors.

### Authors' contributions

EM: development of study design, supervision of data collection and analysis, further interpretation of data, writing and revising manuscript. TGR: development of study design, data collection and analysis, writing and reviewing manuscript. MLH: methodological guidance, further interpretation of data, writing and revising manuscript.

## Appendix

**Table:**

Pharmacist	Practice Manager (if different to pharmacist)	Key Informant (if different to pharmacist)
How long have you been involved in the scheme?		Could you tell me a little bit about your role in the CAS?
Could you share your expectations of the CAS?		
What do you perceive to be the benefits of the CAS for the stakeholders involved? Pharmacist Patient GP		What do you perceive to be the benefits of the CAS for the stakeholders involved? Pharmacist Patient GP LHBs
Could you tell me about any barriers you have encountered to the effective implementation of the CAS?		
The Welsh Assembly Government states that the aim of the CAS is to ‘make community pharmacy the first port of call for the provision of advice and if necessary treatment of common illnesses.’ How far do you feel the CAS has gone into achieving this aim?		
How prepared do you feel to discuss the CAS with patients?		Could you comment on why you think pharmacists have been selected as a first port of call?
How has the CAS affected your working relationships with the local GPs?		
There are two main methods of entry onto the CAS, either the patient can self‐refer themselves, or they can be referred by their GP surgery. Can you tell me your views on these?	Could you talk me through how you refer patients to the CAS?	
Do you have any suggestions on how to improve the CAS?		
Overall, how would you sum up your personal opinion of the scheme in a few sentences?		
Finally, is there anything you would like to add about the service I have not covered?		
Prompts and probes: Can you tell me a little bit more about that? Why is that? Would you be able to comment more on? Do you think there is any reason for that? Could you explain what you mean? What is your opinion on this? Could you give me an example?		
	
